# Therapeutic Management of Probiotics and Prebiotic Products to Modulate Gut Microbiome by Healthcare Services

**DOI:** 10.3390/microorganisms14061360

**Published:** 2026-06-17

**Authors:** Rawan Bajoudah, Li Li, Malik Altaf Hussain

**Affiliations:** School of Science, Faculty of Engineering, Computing and Science, Western Sydney University, Richmond, NSW 2753, Australia

**Keywords:** probiotics, prebiotics, gut microbiome, gut modulation, healthcare professionals

## Abstract

The prescription and recommendation of prebiotics and probiotics by healthcare professionals remain an evolving area of research. Despite increasing recognition of their benefits in modulating the gut microbiome, healthcare professionals including dietitians, often lack standardised guidelines and sufficient training to integrate them into practice. Barriers to implementation include insufficient clinical evidence, cost, uncertainty regarding strain-specific efficacy, and a lack of consensus on appropriate therapeutic applications. Studies indicate that while many healthcare professionals acknowledge the potential benefits of these products, their hesitancy arises from insufficient supporting research evidence and associated education. Future efforts should concentrate on enhancing clinical guidelines, improving educational programs for healthcare professionals, and conducting large-scale trails to establish evidence-based recommendations. Additionally, integrating gut microbiome sequencing into clinical decision-making could improve personalised nutrition and optimise the prescription of prebiotics and probiotics. Further research is necessary to address knowledge gaps and promote effective, evidence-based use of these interventions by healthcare professionals. This review explores current prescribing practices, knowledge, and perceptions of healthcare professionals regarding prebiotics and probiotics with a particular focus on the evidence, education, and implementation gaps that may influence their clinical application.

## 1. Introduction

The human digestive tract contains millions of microorganisms that live in the gastrointestinal tract (GIT) commonly known as the “gut microbiome”. The human gut microbiome plays an important role in health and disease. This complex ecology has an impact on various aspects of human physiology including nutrient metabolism and immune function. Studies have continued providing new insights into the intricate relationship between the gut microbiome, diet, and overall health outcomes. Each individual carries a unique assortment of commensal microbiota with individual-specific functional capabilities [1]. This individual-specific gut ecology has been linked to population-wide heterogeneity in human responses to nutrition, lifestyle, and pharmaceutical interventions.

Maintaining a good balance of the gut microbiome by modulating the bacterial composition, diversity, and activity appears to benefit host health [2]. Modulation of the gut microbiome has emerged as a potentially powerful strategy to enhance the health of the host by protecting against infections and diseases [3]. Strategies such as diet, prebiotics, probiotics, antimicrobial agents, and faecal transplantation have significant potential to modulate and manage the gut microbiome [3]. A recent study highlighted the important role of host–microbiota interactions in cognitive and metabolic health, indicating that modification of the gut microbiome could aid in mitigating disorders such as neurodegenerative diseases and metabolic syndrome [4]. Additionally, targeted precision microbial regulation strategies, including selective probiotic supplementation and dietary interventions, have demonstrated potential in enhancing gastrointestinal and immune health [5]. Furthermore, research indicates that gut microbiota-derived metabolites play a crucial role in immune modulation and overall health, highlighting their potential as therapeutic targets for diseases management [6].

It should be noted that there has been a substantial amount of scientific investigation on the efficacy of probiotics and prebiotics to mitigate various health issues mediated by gut microbiota modulation [7,8,9,10,11,12,13]. Multiple studies have shown that prebiotics, probiotics, and probiotic-driven metabolites known as “postbiotics” have the ability to enhance the balance of gut microbiome composition, maintain the integrity of the gut barrier, and regulate specific immune responses [2]. Additionally, they can prevent the invasion of pathogens and reduce the risks of obesity, type 2 diabetes, inflammatory bowel disease (IBD), cancer, cardiovascular disease, liver disease, and central nervous system disorders [2,14].

Despite the documented health benefits of prebiotics and probiotics, it has been frequently reported that dietitians, nutritionists, and other healthcare professionals lack a good understanding or confidence of their effectiveness as therapeutic agents [8,15,16,17,18,19]. For instance, Rasmussen et al. [8] reported that the majority of healthcare professionals did not recommend prebiotics or probiotics to their clients, although they knew the benefits and effects of using prebiotics and probiotics for health. Similar findings were reported by Oliver et al. [17], who emphasised the need to educate healthcare providers about the use of prebiotics and probiotics to ensure the provision of clinically appropriate recommendations. The development of standardised clinical guidelines for prebiotics and probiotics remains challenging due to factors such as strain-specific effects, variability in clinical outcomes, and limitations in the current evidence base [18,20]. Addressing these challenges may support more consistent recommendations, improve practitioner confidence, and enhance patient outcomes. This review provides an overview of healthcare professionals’ knowledge, perception, and prescribing practices related to prebiotics and probiotics, while highlighting key barriers to their clinical implementation and identifying priorities for future research, education, and evidence-based practice.

## 2. Probiotics and Health

The term ‘probiotics’ was introduced by Werner Kollath in 1953 as “active substances that are essential for a healthy development of life” [21]. Later in 1992, Fuller defined a probiotic as “a live microbial feed supplement which beneficially affects the host animal by improving its intestinal microbial balance” [21,22]. Now, probiotics are defined by the Food and Agriculture Organisation (FAO) and World Health Organisation (WHO) as “live microorganisms which when administered in adequate amounts confer a health benefit on the host” [23]. Even though probiotics have long been utilised by humans, the discovery and development of novel probiotics continue to rely heavily on experiments [24]. These microorganisms, to be classified as probiotics, must meet certain basic criteria that include their ability to survive in acidic pH and high bile salt conditions, having antagonistic effects against pathogens, and function as beneficial agents for the host [25,26]. Several members of the *Bifidobacterium*, *Lactobacillus*, and *Enterococcus* genera are classified as probiotic microorganisms [27]. One main group of probiotic bacteria commonly used in humans and animals is lactic acid bacteria (LAB), including *Bifidobacterium* and *Lactobacillus* [28,29]. The most common species of probiotics that can be found in most probiotic products are listed in Table 1.

Probiotic microorganisms, including bacteria and some yeast, are present naturally in fermented food. Additionally, they can be added to other food products and are also available in dietary supplement form [31]. Probiotics are commonly found in the dairy and dairy-related products, such as yogurt, cultured buttermilk, and cheese, that include LAB and other microorganisms obtained from fermented milk, a useful source of probiotic strains [32,33,34]. Examples of other foods manufactured through microbial fermentation are Japanese miso, tempeh, sauerkraut, beer, sourdough, bread, chocolate, kimchi, olives, pickles, and kefir [34]. In addition, several studies have found the presence of probiotic strains in non-dairy fermented substrates such as soy-based products, cereals, legumes, cabbage, maize, pearl, millet, sorghum, and others [34,35].

Conventional sources of probiotic strains include dairy products, human breast milk, and human faeces, while unconventional sources include non-dairy fermented food products, non-intestinal sources, and various parts of the digestive tracts of various animals [28,36]. Non-dairy probiotics food products are gaining popularity, especially in individuals with lactose intolerance or who want to avoid the consumption of dairy and dairy products [28,36]. Also, products containing probiotic microorganisms are considered functional foods, and they can be added to different foods such as cheese, ice cream, milk-based deserts, butter, mayonnaise, fermented foods of plant origin, fruit juices, vegetables, legumes and cereals, malt, and soybeans [37]. Probiotics are typically incorporated into foods during the fermentation process. However, Nagpal et al. [38] reported that the focus on extending the lifespan of probiotics in food has led to changes in the functionality and effectiveness of the food product. Specifically, probiotic bacteria must maintain viability in food carriers and survive the acidic conditions of the GIT with a minimum count of 10^6^ CFU g^−1^ in the final product, a common challenge during product development [38].

Probiotics offer significant health benefits primarily through the improvement of gastrointestinal microflora health and the management of GIT infections such as diarrhoea, constipation, irritable bowel syndrome, and IBD [25]. Re-establishing the gut microbiota can be challenging, but probiotics have shown positive results in numerous well-designed clinical studies [39]. Probiotics keep the GIT healthy by several mechanisms: (1) maintaining and improving the epithelial barrier; (2) improving intestinal mucosa adhesion and inhibiting pathogen adhesion; (3) competitively excluding pathogenic microbes; and (4) generating anti-microbial substances and immunomodulation action [27,29,40]. Also, probiotics help with digestion and protect the GIT from common pathogenic bacteria such as *Helicobacter pylori* and *Escherichia coli* [27].

Several studies have demonstrated the effectiveness of probiotics in preventing or treating specific health conditions including atopic dermatitis, paediatric acute infectious diarrhoea, antibiotic-associated diarrhoea, IBD, IBS, hypercholesteremia, and obesity [31]. Examples of studies that have shown positive effects of several probiotic strains in patients with different health conditions are given in Table 2. Moreover, probiotic species such as *Lb. bulgaricus* and *Streptococcus thermophiles* are used as possible pharmacological interventions with lactose intolerance patients [41]. The combination of *Lb. casei* Shirota and *B. breve* Yakult demonstrated a more effective impact and considerably alleviated the symptoms of lactose intolerance, as reported by Pandey et al. [41] and Vonk et al. [42].

## 3. Gut Microbiome Modulation Through Probiotics and Prebiotics

**Figure 1:**
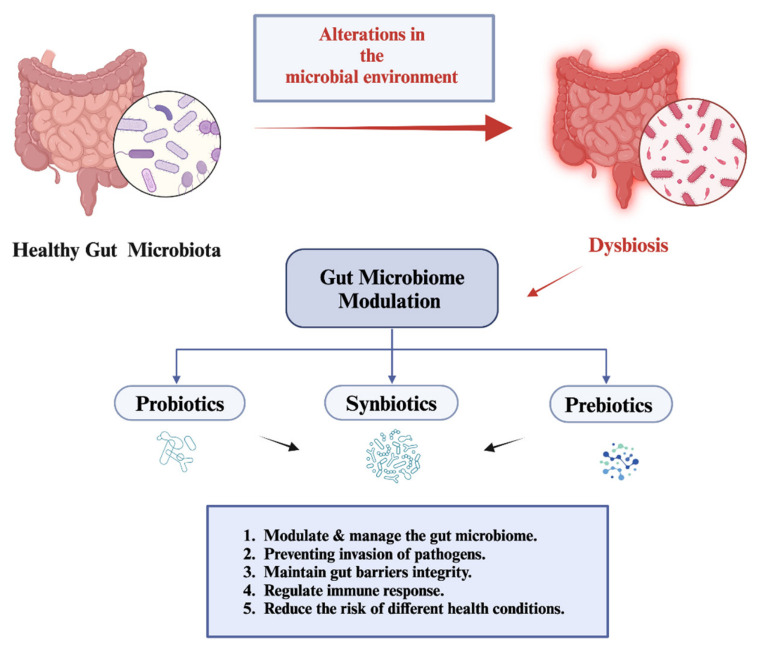
Graphical presentation of the role probiotics and prebiotic in gut microbiome modulation.

The host’s normal physiology relies on the signals provided by the intestinal bacteria found in the intestinal lumen, the initial line of defence against ingested and invasive pathogenic bacteria [39]. The intestinal lumen is comprised of gastric acid, digestive enzymes, immunoglobulin A (IgA), and indigenous bacteria that degrade intraluminal antigens and prevent pathogenic microbes from adhesion and colonization [39,54]. Therefore, Vyas and Ranganathan [39] reported that any alterations in this microbial environment may lead to an imbalance or dysregulation of the microbiota (dysbiosis), which is frequently linked to different disease conditions; thus, it is critical to restore bacterial homeostasis. One of the methods used for the modification of the gut microbiota is the use of prebiotics, probiotics, and synbiotics—a combination of both prebiotics and probiotics [39]. Figure 1 provides an overview of probiotics and prebiotics in modulating the gut microbiome for improved health outcomes.

Probiotics enhance gastrointestinal health by strengthening the intestinal barrier and mitigating gut inflammation, particularly in disorders such as irritable bowel syndrome (IBS) and inflammatory bowel disease (IBD) [55]. In addition to gastrointestinal benefits, probiotics also influence metabolic health by regulating lipid metabolism and enhancing glucose homeostasis, therefore reducing the risk of metabolic syndrome and type 2 diabetes [56]. Moreover, probiotics have demonstrated the ability to modulate immune function by enhancing anti-inflammatory responses and promoting the synthesis of beneficial microbial metabolites that help maintain immune homeostasis [57]. Recent evidence suggests that probiotics also contribute to neurological health, since interactions between gut microbiota and the central nervous system may mitigate symptoms of anxiety and depression through modulation of the gut–brain axis [58]. These findings highlight the increasing recognition of probiotics as a treatment strategy for various physiological systems beyond the GIT.

Furthermore, a combination of prebiotics and probiotics can influence the microbiota directly or indirectly, which makes them beneficial therapies for enhancing human health [59]. The field of microbiomics has led to a significant rise in scientific, industrial, and public interest in probiotics and prebiotics as potential tools for managing and controlling gut microbiota [39,60,61,62]. However, choosing the appropriate type of probiotics is challenging for healthcare professionals and also the public [20]. ‘Strain specificity’ and ‘disease specificity’ are two important factors that should be considered by the healthcare providers when prescribing the appropriate type of probiotics for their patient. McFarland et al. [20] affirmed that clinical guidelines and meta-analyses should emphasise the importance of reporting outcomes based on the specific strains of probiotics and the specific type of disease to analyse the efficacy and safety of probiotics. Examples of probiotics and prebiotics recommendations based on the results of using advanced omics technologies are given in Table 3.

The concept of prebiotics was presented for the first time in 1995 by Gibson and Roberfroid [63], and they defined it as “nondigestible food ingredients that beneficially affect the host by selectively stimulating the growth and/or activity of one or a limited number of bacterial species already resident in the colon, and thus attempt to improve host health”. Davani-Davari et al. [64] reported that for over 15 years, this definition had remained, and according to this, only a few compounds in the carbohydrate group can be classified as prebiotics. Advancements in microbiome research and high-throughput sequencing tools have increased the understanding of the gut microbiota’s complexity and its interaction with the host, leading to an expanded concept and definition of prebiotics [65,66]. Later in 2008, dietary prebiotics were defined in the 6th Meeting of the International Scientific Association for Probiotics and Prebiotics (ISAPP) as “a selectively fermented ingredient that results in specific changes in the composition and/or activity of the gastrointestinal microbiota, thus conferring benefit(s) upon host health” [64,67]. In 2016, an ISAPP expert panel updated the definition of a prebiotic, which was subsequently published in 2017 as a “a substrate that is selectively utilized by host microorganisms conferring a health benefit” [68].

**Table 3:** Prebiotics and probiotics recommendations based on omics sequencing.

Omics Sequencing Result	Prebiotic Recommendation	Probiotic Recommendation	References
Low Diversity Microbiome	Inulin FOS GOS	*Lactobacillus* species *Bifidobacterium* species	[64,69,70]
Low *Bifidobacteria* Levels	FOS GOS AXOS Polyphenols	*Bifidobacterium* species *Lactobacillus* species	[70,71,72,73,74]
Low *Lactobacillus* Levels	FOS GOS Polyphenols	*Lactobacillus* species *Bifidobacterium* species	[70,71,73,75]
High *Firmicutes* to *Bacteroidetes* Ratio	Resistant starch Beta-glucans	*Lactobacillus* *Bacillus* Yest from the genus *Saccharomyces*	[76,77,78]
High Proteobacteria Levels	Polyphenols	*S. boulardii* *Lb. reuteri* *Lb. fermentum*	[70,79,80,81]

Abbreviations: FOS, fructo-oligosaccharides; GOS, galacto-oligosaccharides; AXOS, Arabinoxylan-oligosaccharides.

Prebiotics are short-chain carbohydrates (SCCs), non-digestible by human digestive enzymes, and selectively enhance the function of certain beneficial bacteria [82]. Prebiotics pass from the small intestine to the lower gut, where they are metabolised by probiotic bacteria without being utilised by other bacteria in the intestinal tract [82]. In the human intestine, the enzymes essential to breaking down the polymeric bonds of prebiotics are usually not present, and this allows the prebiotics to pass through the small intestine without being digested and reach the colon, where they are fermented by the potentially beneficial bacteria as *Lactobacillus* and *Bifidobacterium* [83,84]. The beneficial gut bacteria ferment these compounds in the intestine to produce short-chain fatty acids (SCFAs), including acetic acid, propionic acid, and butyric acid, which are critical end components of carbohydrate metabolism [82,85,86]. These SCFAs serve as an energy source for the host organism [82]. The production of SCFAs is linked to several physiological benefits, such as enhanced intestinal function, mineral absorption, lipid regulation, glucose metabolism, and reducing the risk of colon cancer [86].

Prebiotic intake promotes the growth of beneficial bacteria such as *Lactobacillus* and *Bifidobacterium* and inhibits the growth of pathogenic bacteria, thereby aiding in the prevention of infections and allergies [86,87]. As a result, this leads to improved intestinal membrane integrity and nutrient absorption; a lower glycaemic level and body weight; the inhabitation of carcinogen toxicity; improved immunity; and the modulation of metabolic, cardiovascular, and inflammatory biomarkers [86,88]. In addition, the intake of prebiotics may lead to an improvement in health issues associated with dysbiosis or even a rebalance of the intestinal microbiota [86,89]. It is reported by Maguire and Maguire [90] and Khangwal and Shukla [89] that several experimental studies have proven that prebiotics can reduce the severity of particular diseases such as diabetes, irritable bowel syndrome, neural disorders, and other infectious diseases. Some examples of studies that showed the effect of prebiotics on gastrointestinal disorders, including irritable bowel syndrome (IBS), Crohn’s disease (CD), colorectal cancer (CRC), and ulcerative colitis (UC), are described in Table 4.

**Figure 2:**
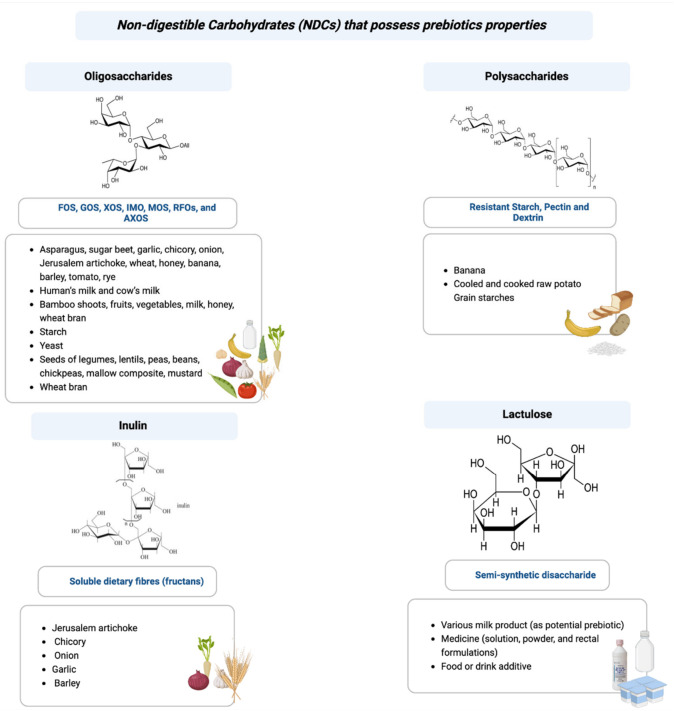
Examples of non-digestible carbohydrates that possess prebiotics properties and their food sources.

Non-digestible carbohydrates can be classified as prebiotics if they achieve the following criteria: resistance to gastric acidity and mammalian enzymes; susceptibility to fermentation by gut bacteria; and the capacity to improve the viability and/or activity of beneficial microorganisms [82,95]. Non-digestible carbohydrates that are considered food components with prebiotic properties and their sources are listed in Figure 2. The majority of prebiotics are a subset of carbohydrate groups, primarily oligosaccharide carbohydrates [64]. Prebiotics that are typically used in the human diet include Lactulose, galacto-oligosaccharides (GOS), fructo-oligosaccharides (FOS), inulin and its hydrolysates, maltooligosaccharides, and resistant starch [82]. In particular, only bifidogenic, non-digestible oligosaccharides such as inulin, FOS, and (trans) GOS meet all the requirements for prebiotic classification [41,96].

A healthy diet is described as one that contains all the essential nutrients and prebiotics that can help prevent diseases associated with lifestyle by promoting the growth of the beneficial gut microflora in the large intestine [89]. Prebiotics are naturally found in many foods such as asparagus, garlic, chicory, onion, Jerusalem artichoke, human and cow’s milk, unrefined wheat and barley, raw oat, rye, honey, sugar beetroot, banana, tomato, soybeans, peas, beans, seaweed, microalgae, and non-digestible carbohydrates (particularly non-digestible oligosaccharides) [41,64]. Certain prebiotics, including inulin, are classified as low digestible carbohydrates and have been linked to impaired gastrointestinal tolerance, particularly when consumed in large amounts [97,98]. Other prebiotic fibres, such as wheat dextrin and polydextrose, demonstrate high tolerance in the GIT [98,99].

Prebiotics are also available as prebiotic supplements to promote the growth of beneficial intestinal microbes, and this could be a useful nutritional therapy in managing metabolic abnormalities and chronic diseases [66]. Prebiotics supplements are produced on large scales because it is available in foods in low concentrations [64]. Prebiotics have been widely utilised by the food industry in various preparations as functional ingredients due to their beneficial effects on human health [86,87]. For instance, prebiotics can be used in producing dairy products, confectionery, infant formulas, whole wheat bread, cereal bars, chocolate, meat products, and other products [86,100].

## 4. Therapeutic Management of Probiotics and Prebiotics by Healthcare Services

The knowledge, perception, and use of probiotics and prebiotics among dietitians, nutritionists, and other healthcare professionals have been previously assessed [8,15,16,17,18]. Rasmussen et al. [8] assessed the knowledge, use, and perception of probiotics and prebiotics among healthcare service providers and found that 97% of them were familiar with probiotics, whereas only 28% were familiar with prebiotics. Furthermore, 63% and 55% of healthcare professionals, respectively, believed that probiotics and prebiotics contribute to general health; however, the majority had never advised patients to take probiotics (57%) or prebiotics (75%). Similarly, Oliver et al. [17] reported that healthcare service providers were more familiar with the term probiotic than prebiotics. Despite their recognition of potential health benefits, 55% had never recommended probiotics, and 74% had never recommend prebiotics to patients [17]. These findings underscore the need for enhanced education among healthcare providers to ensure evidence-based recommendations for probiotics and prebiotics.

Flach et al. [18] conducted a study involving 208 general practitioners and 207 medical specialists to review medical doctors’ perspectives on probiotics and their efficacy. Their findings indicated that 51% of participants recommended probiotics, primarily for antibiotic-associated diarrhoea (74%) and IBS (51%). However, 53% refrained from recommending probiotics due to a perceived lack of scientific evidence. The study highlighted the need for large-scale controlled trials and comprehensive clinical guidelines to improve medical doctors’ perceptions of probiotics and facilitate their integration into clinical practice. Similarly, Fijan et al. [15] conducted a cross-sectional study to evaluate health professionals’ knowledge and beliefs regarding probiotics. They found that 36.4% of respondents rated their knowledge of probiotics as moderate, 36.2% as good, and only 8.9% as excellent. Pharmacists, allied health professionals, and other healthcare professionals reported higher self-rated knowledge compared to medical doctors and nurses. The study concluded that targeted educational programs are essential to enhance health professionals’ understanding and the use of probiotics in clinical settings.

Valdovinos-García et al. [16] examined probiotics prescription practices among gastroenterologists and nutritionists. Their findings showed that 64.9% of participants always recommended probiotics, 31.7% rarely recommended them, and 3.6% never recommended them. Gastroenterologists primarily prescribed probiotics for treating illnesses (56.5%), while nutritionists recommended them for maintaining general health (39%). Moreover, 97% of gastroenterologists and 98% of nutritionists considered probiotics safe and effective for managing gastrointestinal symptoms. Despite these positive perceptions, the study emphasised that probiotics efficacy in digestive disorders remains partially known due to a lack of scientific evidence and therapeutic guidelines, underscoring the need for continued medical education and standardised clinical recommendations.

In addition to previous findings, Mabonga et al. [101] investigated the role of probiotics in personalised cancer therapy, particularly for managing chemotherapy and immunotherapy-induced gut dysbiosis. The study showed that oncologists remain hesitant to recommend probiotics due to insufficient findings from clinical trials involving cancer patients and concerns over safety in immunocompromised individuals. Although probiotics show potential in mitigating treatment side effects, the absence of large-scale randomised controlled trails (RCTs) and oncology-specific guidelines restricts their clinical application. The study highlighted the need for further research to develop clear recommendation for probiotic use in cancer treatment.

Similarly, Pettoello-Mantovani et al. [102] conducted a cross-sectional study among paediatric healthcare professional in Europe to assess their knowledge and use of probiotics. Their findings demonstrated that many paediatricians lacked sufficient knowledge regarding probiotic strains and their specific clinical applications, leading to reluctance in recommending them for paediatric gastrointestinal disorders. While some paediatricians actively prescribed probiotics, other cited insufficient clinical evidence, and the absence of standardised guidelines as major barriers. The study underscores the importance of integrating probiotic education into paediatric medical training to equip healthcare professionals with the knowledge required for informed recommendations.

Moreover, Yahya [103] investigated the knowledge, attitudes, and practices of dermatologists and family physicians in Saudi Arabia regarding the use of probiotics for targeting atopic dermatitis. The study found that only 33.3% of practitioners actively monitored patient outcomes after prescribing probiotics, while the majority refrained from routine recommendations due to insufficient clinical evidence supporting their efficacy in dermatological conditions. The findings highlight the need for additional research on probiotics in dermatology and the development of clear therapeutic guidelines to facilitate their broader adoption among healthcare professionals.

Abbas et al. [104] examined pharmacists’ knowledge, perceptions, and prescribing practices of probiotics in the UAE. The study found that many pharmacists hesitated to recommend probiotics due to high cost, the absence of standardised clinical guidelines, and inadequate awareness among healthcare professionals. Pharmacists also reported difficulties in determining appropriate probiotic dosages and strain selection due to limited training. Abbas et al. [104] suggested that standardised clinical guidelines and targeted professional education programs for pharmacists could enhance the rate of probiotics prescriptions. While it seems that practitioners may need further training about the use of pre- and probiotic products, public awareness and the adoption of probiotics and prebiotics are increasing, as stated by Cunningham et al. [60] and Chin-Lee et al. [105]. Specifically, the probiotic market is predicted to grow by 7% annually, while prebiotic growth is forecasted to 12.7% over the next 8 years [60,106,107]. Despite the abundant and widely accessible evidence suggesting probiotics use, people are still unfamiliar with probiotics [25]. Therefore, Islam et al. [25] suggested that it is necessary to change people’s attitudes towards the use of probiotics because they found their knowledge about the health benefits is limited. Consumer knowledge and experience with probiotics and prebiotics have been documented by several authors [25,108,109,110,111,112,113] (Table 5).

**Table 5:** Key challenges in translating gut microbiome science into dietetic practice and their implications for clinical applications.

Gap	Why It Matters in Practice	What Is Needed
Limited translation of microbiome research into clinical practice	Research findings are difficult to apply in real clinical settings	More focus on clinically relevant outcomes and practical application
Challenges in interpreting microbiome data	Difficulty in making consistent and evidence-based decisions	Structured frameworks and decision-support tools
High inter-individual variability	Reduced consistency and predictability of interventions	Context-specific approaches that consider individual patient factors
Lack of standardised guidance in Australia	Variability in practice and uncertainty in probiotic use	Development of clear, evidence-based national guidance
Increasing consumer use of microbiome testing and probiotics	Inconsistency between consumer expectations and current evidence	Improved communication and guidance from dietitians

An Australian study investigated the awareness and attitudes of Australian adults about gut health, probiotics, and factors associated with probiotic use [109]. They found that 58.9% of the participants were probiotics users. The participants with a healthier lifestyle and a better understanding of gut health and probiotics were more likely to use probiotics. Additionally, it was reported that 59% of participants who were not using probiotics showed a willingness to try probiotics based on recommendations from healthcare professionals. They recommended that provision of education on gut health and probiotics by health professionals might enhance probiotic use, particularly in populations that are more likely to gain advantages, such as individuals with a specific condition or unhealthy lifestyle.

The investigation on the use probiotics by IBD patients found that probiotics were used for managing IBD in 82% of CD patients and in 88% of UC patients [110]. It was found that IBD patients who used probiotics to control their health chose ineffective strains for their condition as they selected them without a prescription from medical providers. Additionally, Hedin et al. [110] stated that IBD patients frequently rely on nonclinical sources of information regarding the use of probiotic products. Thus, Hedin et al. [110] highlighted the importance of monitoring patients’ probiotic use by their healthcare practitioners and providing them with evidence-based advice.

A recent study investigated the level of interest in prebiotics among consumers in Romania and found that 74% of participants were familiar with prebiotics and 25% were not [65]. They found that 29.1% of participants believed that consuming foods with compounds that may have prebiotic effects improved digestion, while 21.3% thought it supported the immune system, 16.8% thought it contributed to better nutrient absorption, and 15.6% thought it had detoxifying properties. The findings of the study clearly demonstrate consumers’ limited knowledge of prebiotics, which warrants healthcare providers to provide more comprehensive information about the relationship between food consumption and health outcomes to their patients.

Moreover, an investigation on the probiotics use, beliefs, and experiences in patients with cystic fibrosis (CF) in Australia found that 73% of participants used probiotics primarily to manage gastrointestinal- and antibiotic-related issues [113]. Additionally, they found that 73% of participants did not talk about their probiotic use with their clinicians, and 33% were unsure if using probiotics had been beneficial. Anderson, et al. [113] highlighted the importance of further research on the effects of probiotics on gastrointestinal issues, as it will provide valuable insights for recommendations regarding their use in CF patients and other chronic diseases.

The studies reviewed here collectively indicate that although healthcare professionals recognise the potential health benefits of probiotics and prebiotics, their knowledge, prescribing practices, and confidence in these interventions remain limited. The absence of standardised clinical guidelines, insufficient numbers of large-scale well-designed clinical trials, inadequate professional education, confusion due to strain-specific effects, and cost considerations contribute to practitioners’ hesitancy to routinely recommend probiotics and prebiotics. These barriers have been reported across a range of healthcare professionals, including medical doctors, pharmacists, and paediatricians, who have expressed concern about the efficacy and appropriate use of probiotics due to the absence of standardised recommendations.

To address these barriers, key strategies such as comprehensive professional education, the development of standardised clinical recommendations, and increased funding for large-scale randomised controlled trials are necessary. Furthermore, integrating probiotics and prebiotics education into medical and healthcare training programs may enhance awareness and improve practitioners’ confidence in prescribing probiotics and prebiotics. Professional development workshops, continuing education programs, and evidence-based guidance developed by professional organisations may further support the consistent implementation of probiotics and prebiotics in clinical practice.

Another significant gap identified in the literature is the lack of studies investigating the prescription of probiotics and prebiotics based on the gut microbiome sequencing services. Most research has focused on the general use and effects of probiotics and prebiotics for specific health conditions [15,109,113]. Therefore, further research is necessary to investigate dietitians and other health practitioners’ experiences with prescribing probiotics and prebiotics based on gut microbiome sequencing services to better understand current practices and potential development in this area.

## 5. Emerging Applications and Future Opportunities

Advancement in biotechnology, synthetic biology, and food processing technologies are shaping emerging applications and future opportunities for probiotics and prebiotics. Traditional probiotics, including *Lactobacillus* and *Bifidobacterium* species, have established the basis for microbiome-targeted therapeutics. However, the discovery of next-generation probiotics (NGPs), such as *Roseburia intestinalis*, *Faecalibacteruim prausnitzii*, and *Akkermansia muciniphila*, has expanded therapeutic options [60]. These species, identified through using genome sequencing and cultivation techniques, are known to produce SCFAs essential for gut health, immune modulation, and metabolic regulation [60,114,115,116]. For instance, *A. muciniphila* has demonstrated potential in enhancing glucose metabolism and preventing obesity, with human studies validating its safety and effectiveness in both live and pasteurised forms [60,117,118]. Even with these advancements, there are still challenges to increase the production of these probiotics because, primarily due to their need for anaerobic growth conditions and the related manufacturing expenses [60].

Prebiotics have similarly advanced beyond their traditional role of selectively targeting *Lactobacilli* and *Bifidobacteria*. Recent findings emphasise their capacity to stimulate a broader range of beneficial taxa, including *Christensensella* spp. and *Propionibacterium* spp., both of which are known to produce SCFAs [60,68]. Emerging prebiotic candidates include plant-based carbohydrates, human milk oligosaccharides (HMOs), yeast-derived compounds, and polyphenols, such as those from cranberry extracts that selectively enhance *A. muciniphila* [60,119,120]. Furthermore, by-products generated from food production, including pectin derived from orange peels and arabinoxylans sourced from brewing waste, represent sustainable and cost-effective prebiotic sources [60,121,122].

Food processing technologies such as high-pressure processing, cold plasma, and ultrasound are advancing the development of probiotics and prebiotics. These techniques maintain bioactivity and enhance product stability and functionality [123]. Synthetic biology facilitates the engineering of probiotics to detect environmental signals and generate therapeutic proteins, which can be applied in the management of metabolic disorders, including diabetes and phenylketonuria [124]. The integration of engineered probiotics with prebiotic substrates can improve their viability and functionality, establishing a foundation for next-generation synbiotics [124,125].

Future research will likely increasingly emphasise personalised approaches that utilise machine learning and bioinformatics to tailor synbiotic interventions according to individual microbiome profile [60,126]. Prebiotics are being investigated for their ability to modulate microbiomes in other areas beyond the gut, such as the skin and the oral cavity. For example, glucomannan hydrolysates have shown efficacy in enhancing the skin microbiome and mitigating acne when applied topically [60,127]. As of 2020, more than 245 clinical trials have been completed, providing substantial evidence supporting the efficacy of using probiotics and prebiotics in managing conditions such as IBS, autism, and obesity, highlighting their growing role in health care [60,118,128].

Finally, these technological innovations equip healthcare providers with tools to prescribe probiotics and prebiotics more effectively by addressing key challenges in functionality, stability, and therapeutic accuracy. Synthetic biology facilitates the development of probiotics specifically engineered to target conditions such as metabolic disorders and inflammation, while selective prebiotic substrates can improve their violability in the gut. Novel prebiotic sources, such as sustainable by-products from food production and polyphenols, expand the range of microbiome-modulating options for clinical application. Moreover, advancements in processing technologies, including ultrasound and high-pressure processing, ensure that these products maintain their bioactivity and efficacy, even in adverse storage and delivery conditions. By leveraging these innovations, healthcare providers can offer targeted and personalised microbiome-based interventions, improving patient outcomes across a variety of conditions.

## 6. Conclusions

In conclusion, although probiotics and prebiotics have been widely recognised for their health benefits, their application by healthcare professionals remains inconsistent due to limited guidelines, training, and scientific consensus. Enhancing clinical guidelines, expanding professionals’ education, and advancing research on microbiome-based prescribing can enhance confidence in their therapeutic applications. Future research should focus on incorporating gut microbiome analysis into healthcare professionals’ practice, ensuring more precise and personalised probiotics and prebiotics recommendations. Additional research is needed to conduct large-scale clinical trials, evaluate the clinical utility of microbiome sequencing services, and develop evidence-based clinical frameworks. Educational strategies are also needed to improve healthcare professionals’ knowledge and confidence in microbiome-informed interventions.

## Figures and Tables

**Table 1:** Common species of probiotics.

Probiotic Genus	Representative Species	Reclassified Name ^1^	References
*Bifidobacterium*	*Bifidobacterium longum* *B. breve* *B. bifidum* *B. infantis*		[27]
*Lactobacillus*	*Lactobacillus plantarum* *Lb. rhamnosus* *Lb. coryniformis* *Leuconostoc mesenteroides* *Lb. brevis* *LB. acidophilus*	*Lactiplantibacillus plantarum* *Lacticaseibacillus rhamnosus* *Levilactobacillus brevis*	[25,30]
*Enterococcus*	*Enterococcus faecalis* *E. faecium*		[27]
Other lactic acid bacteria	*Lactococci* *Streptococci*		[24]
Other probiotic strains (organisms of the bacterial genera)	*Bacillus* *Escherichia* *Propionibacterium* *Saccharomyces*		[24]

^1^ The species were reclassified based on several genetic approaches and markers.

**Table 2:** Examples of studies demonstrate positive health effects of probiotics strains.

Health Condition	Sample Size	Probiotic Type	Dose	Effect	Reference
Inflammatory Bowel Disease	Adults with UC (*n* = 65)	VSL#3	3600 billion CFU/day for 8 weeks	▪ Reduce in UC activity index and improve rectal bleeding	[43]
Pediatric Acute Infectious Diarrhea	Children (*n* = 186)	*Saccharomyces boulardii*	200 mg 2/day for 5 days	▪ Reduction in diarrhea duration	[44]
Antibiotic-Associated Diarrhea	Children (*n* = 167)	*S. boulardii*	500 mg/day + AB for 2 weeks	▪ Reduce diarrhea prevalence and increase in the recovery rate	[45]
Obesity	Adults with large visceral fat areas (*n* = 210)	Fermented milk containing a probiotic; *Lb. gasseri*	200 g/day for 12 weeks	▪ Decrease in abdominal visceral fat area ▪ No change in the abdominal subcutaneous fat area ▪ Decrease in BMI, waist and hip circumferences, and body fat mass measures	[46]
	Adults (*n* = 100)	SK0-001; *L.* *plantarum*	1 capsule/day for 12 weeks	▪ Decrease in body fat percentage, mass, and LDL-C ▪ Increase in adiponectin levels	[47]
Atopic Dermatitis	Children (*n* = 43)	*Lb. salivarius* LS01	2 sachets/day for 8 weeks, and 1 sachets/day for the following 8 weeks	▪ Significant decrease in SCORAD/objective, SCORAD index and itch intensity	[48]
Irritable Bowel Syndrome	Adults (*n* = 152)	Symprove; *Lb. rhamnosus*, *Lb. plantarum*, *Lb. acidophilus*, and *Enterococcus faecium*	1 mL/kg/day for 12 weeks	▪ Significant improvement in overall symptom severity	[49]
Hypercholesterolemia	Adults (*n* = 31)	*Lb. acidophilus*, and *B. bifidum*	3 capsules/day for 6 weeks	▪ Decrease serum total cholesterol, LDL, and HDL ▪ No effect on serum triglyceride or fasting blood glucose levels	[50]
Cognitive function	Adults (*n* = 200)	*Lb. rhamnosus GG*	10 billion CFU/day for 3 months	▪ Improve in total cognitive score in individual with cognitive impairment ▪ No significant effect in in those without cognitive impairment	[51]
Polycystic Ovary Syndrome	Adults (*n* = 60)	*Lb. acidophilus*, *Lb. casei*, *and B. bifidum*	2 × 10^9^ CFU/g daily	▪ Decrease in serum total testosterone levels and in high-sensitivity C-reactive protein (hs-CPR) levels ▪ Reduction in hirsutism (lower mF-G scores) and malondialdehyde (MDA) concentrations ▪ Increase in sex hormone-binding globulin (SHBG) levels ▪ Increase in total antioxidant capacity (TAC)	[52]
Relapsing-Remitting Multiple Sclerosis	Adults (*n* = 40)	*Saccharomyces boulardii*	250 mg daily for 16 weeks	▪ Improve mood, reduce anxiety and depression ▪ Enhance physical and mental well-being ▪ Decrease fatigue and pain severity ▪ Lower inflammation and oxidative stress markers	[53]

Abbreviations: AB, Intervention antibiotics; UC, ulcerative colitis; VSL#3, high-potency probiotic mixture; LDL, low-density lipoprotein; HDL, high-density lipoprotein; BMI, body mass index.

**Table 4:** Example of previous studies on the effects of prebiotics on gastrointestinal disorders.

Gastrointestinal Disorders	Prebiotic Type	Effect	References
Irritable Bowel Syndrome	scFOS: 5 g/day for 4 weeks	▪ Stimulate the growth of *Bifidobacterium* ▪ Reduce the intensity of IBS symptoms ▪ Decrease in anxiety and depression ▪ No improvement in rectal sensitivity	Azpiroz, et al. [91]
Crohn’s Disease	FOS: 15 g/day for 3 weeks	▪ Increase faecal bifidobacterial concertation ▪ Decrease CD activity ▪ Alter the function of mucosal dendritic cells	Lindsay, et al. [92]
Colorectal Cancer	FOS, XOS, polydextrose, and resistance dextrin: 30 g/day for 1 week	▪ Significant effect on immunological indices ▪ Increase the levels of immunoglobulin G, IgM, and transferrin ▪ Increase the abundance of Bifidobacterium and Enterococcus ▪ Decrease the abundance of Bacteroides	Xie, et al. [93]
Ulcerative Colitis	GOS: 2.8 g/day for 6 weeks	▪ Increase the abundance of *Bifidobacterium* and *Christensenellaceae* ▪ Increase the normal stool proportion (BSFS) ▪ Reduce the incidence and severity of loose stool (GSRS) and urgency (SCCAI)	Wilson, et al. [94]

Abbreviations: scFOS, short-chain Fructooligosaccharides; FOS, Fructooligosaccharides; XOS, Xylooligosacharides; GOS, Glactooligosaccharides.

## Data Availability

No new data were created or analyzed in this study. Data sharing is not applicable to this article.
